# Role of biostimulants in mitigating the effects of climate change on crop performance

**DOI:** 10.3389/fpls.2022.967665

**Published:** 2022-10-21

**Authors:** Ingudam Bhupenchandra, Sunil Kumar Chongtham, Elangbam Lamalakshmi Devi, Ramesh R., Anil Kumar Choudhary, Menaka Devi Salam, Manas Ranjan Sahoo, Tshering Lhamu Bhutia, Soibam Helena Devi, Amarjit Singh Thounaojam, Chandana Behera, Harish. M. N., Adarsh Kumar, Madhumita Dasgupta, Yumnam Prabhabati Devi, Deepak Singh, Seema Bhagowati, Chingakham Premabati Devi, Hemam Ramananda Singh, Chingakham Inao Khaba

**Affiliations:** ^1^ Indian Council of Agricultural Research (ICAR)–Krishi Vigyan Kendra Tamenglong, Indian Council of Agricultural Research (ICAR) Research Complex for NorthEastern Hill (NEH) Region, Manipur Centre, Imphal, Manipur, India; ^2^ Multi Technology Testing Centre and Vocational Training Centre, College of Agricultural Engineering and Post Harvest Technology (CAEPHT), Central Agricultural University (CAU), Ranipool, Sikkim, India; ^3^ Indian Council of Agricultural Research (ICAR)-Research Complex (RC) for North Eastern Hill (NEH) Region, Sikkim Centre, Tadong, Sikkim, India; ^4^ Division of Plant Physiology, Indian Council of Agricultural Research (ICAR)–Indian Agricultural Research Institute, New Delhi, India; ^5^ Division of Agronomy, Indian Council of Agricultural Research - Indian Agricultural Research Institute, New Delhi, India; ^6^ Division of Crop Production, Indian Council of Agricultural Research - Central Potato Research Institute, Shimla, India; ^7^ Amity University Uttar Pradesh, Noida, India; ^8^ Central Horticultural Experiment Station, Indian Council of Agricultural Research (ICAR)–Indian Institute of Horticultural Research, Bhubaneswar, Odisha, India; ^9^ Department of Crop Physiology, Assam Agricultural University, Jorhat, Assam, India; ^10^ Medicinal and Aromatic Plants Research Station, Anand Agricultural University, Anand, Gujarat, India; ^11^ Department of Plant Breeding and Genetics, College of Agriculture, OUAT, Bhawanipatna, India; ^12^ Indian Council of Agricultural Research (ICAR)–Indian Institute of Horticultural Research, Farm Science Centre, Gonikoppal, Karnataka, India; ^13^ Indian Council of Agricultural Research: National Bureau of Agriculturally Important Microorganism, Mau, India; ^14^ Indian Council of Agricultural Research (ICAR)–Research Complex for NorthEastern Hill (NEH) Region, Manipur Centre, Imphal, Manipur, India; ^15^ Indian Council of Agricultural Research (ICAR)-Krishi Vigyan Kendra, Chandel, Indian Council of Agricultural Research (ICAR) Research Complex for NorthEastern Hill (NEH) Region, Manipur Centre, Imphal, Manipur, India; ^16^ Krishi Vigyan Kendra Bhopal, Indian Council of Agricultural Research (ICAR) Central Institute of Agricultural Engineering, Bhopal, Madhya Pradesh, India; ^17^ Department of Soil Science, Assam Agricultural University, Jorhat, Assam, India; ^18^ Krishi Vigyan Kendra, Central Agricultural University (CAU), Imphal, Manipur, India; ^19^ Department of Plant Pathology, Assam Agricultural University, Jorhat, Assam, India

**Keywords:** biostimulant, climate change, drought, temperature, salinity, food security

## Abstract

Climate change is a critical yield–limiting factor that has threatened the entire global crop production system in the present scenario. The use of biostimulants in agriculture has shown tremendous potential in combating climate change–induced stresses such as drought, salinity, temperature stress, etc. Biostimulants are organic compounds, microbes, or amalgamation of both that could regulate plant growth behavior through molecular alteration and physiological, biochemical, and anatomical modulations. Their nature is diverse due to the varying composition of bioactive compounds, and they function through various modes of action. To generate a successful biostimulatory action on crops under different parameters, a multi–*omics* approach would be beneficial to identify or predict its outcome comprehensively. The ‘*omics’* approach has greatly helped us to understand the mode of action of biostimulants on plants at cellular levels. Biostimulants acting as a messenger in signal transduction resembling phytohormones and other chemical compounds and their cross–talk in various abiotic stresses help us design future crop management under changing climate, thus, sustaining food security with finite natural resources. This review article elucidates the strategic potential and prospects of biostimulants in mitigating the adverse impacts of harsh environmental conditions on plants.

## 1 Introduction

Climate is the most crucial factor that significantly influences crop production and productivity, thereby threatening the sustainability of crop production systems and global food security (Swaminathan and Keshvan, 2012). Frequent manifestations of extreme events of drought, heat waves and floods, high and low temperatures, and salinity are among the most common existing stresses in agriculture, directly or indirectly impacting crop production. By the end of the 21st century, the mean global temperature may surge by another 1.5 to 2°C (Anonymous, 2022). This climate change scenario is a severe concern for developing countries like India, where 2/3rd of the arable land is rainfed (Venkateswarlu and Singh, 2015; Datta et al., 2022). The earth has experienced augmented rainfall with diminishing rainy days, showing tremendous rainfall variability in the last four decades (Anonymous, 2022). In India, about 147 million hectares of land are prone to soil degradation, which comprises water erosion (94 m ha), salinity (23 m ha), water–logging (14 m ha), wind erosion (9 m ha), and other forces (7 m ha) (Kumar and Sharma, 2020). Therefore, an integrated and sustainable way of crop production could be one of the possible ways to meet the present and future food security in the context of climate change. Adaptation and mitigation strategies became a research focus to ameliorate the climate–change effects on agriculture through various technological interventions to restore the ecology.

Biostimulants (BSts) could be a promising tool in the current crop production scenario. BSt is a compound or mixture of various organic compounds of natural origin that can promote plant growth under various environmental stresses (Bhupenchandra et al., 2020; Rouphael and Colla, 2020). According to the definition given by the Fertiliser (Inorganic, Organic or Mixed) (Control) Amendment Order, 2021, the Government of India BSt means a “substance or microorganism or an amalgamation of both whose primary function is to enhance physiological processes in plants and to augment its nutrient uptake efficiency, growth, yield, quality, and tolerance to stresses. It does not encompass pesticides or plant growth regulators governed under the Insecticide Act of 1968. It can be broadly classified into various categories such as botanical extracts (including seaweed extracts), protein hydrolysates, amino acids, vitamins, cell–free microbial products, antioxidants, anti–transpirants, humic and fulvic acid, and their derivatives” (Khurana and Kumar, 2022). BSts differ from manures and fertilizers according to their usage in minute quantities (Zhang and Schmidt, 1999; du Jardin, 2015). They could help maintain the ecological balance of agroecosystems, reducing the usage of pesticides and chemical fertilizers or heavy metals for agricultural practices. Considering its immense potential, the European Commission has set a goal to replace 30% of chemical fertilizers with organic–based inputs by the end of 2050 (Hansen, 2018). The use of BSts is increasing and expanding rapidly at a remarkable rate in the present scenario concerning sustainable ecosystems and food security. Jing et al. (2022) reported an increase of 17.9% in yield using BSts in cereals, legumes, fruits, and vegetables. In addition to augmenting the production level, BSts have demonstrated the potential to reduce the release of greenhouse gases through reduced fertilizer consumption in agriculture. Singh et al. (2018) noted that applying *Kappaphycus alvarezii* seaweed extract in sugarcane cultivation could reduce greenhouse gas emissions by supplementing synthetic fertilizer input. They observed a potential saving of 260 kg CO_2_ equivalent/Mg cane production/ha through 5% foliar application of *Kappaphycus alvarezii* seaweed extract and the recommended fertilizer rate. Similarly, Hamedani et al. (2020) reported a marked reduction in CO_2_ equivalent emission in zucchini and spinach cultivation at the tune of 7–12% and 7–24%, respectively, due to the use of mycorrhizal fungus *Glomus intraradices* and vegetal–derived protein hydrolysate (Trainer®, Italpollina S.p.A., Rivoli Veronese, Italy). The development of BSts from organic by–products could aid in better waste management by preventing unplanned discarding and providing ample scope for waste reuse. (Xu and Geelen, 2018). The production of seaweed–based BSts is more eco–friendly than synthetic fertilizers (Ghosh et al., 2015; Anand et al., 2018; Singh et al., 2018).

BSts contain various fractions of bioactive components; hence the study of synergistic effects of all the components on plant growth is tedious. BSt–induced responses vary from morphological modifications to phyto–hormone responses to gene expression. The response also differs among the plant species and their mode of application. This paper aims to highlight the extraordinary potential and scope of BSt for mitigating the adverse abiotic stress, viz., drought, salinity, and temperature stress induced by climate change without compromising crop production, productivity, and quality aspects.

## 2 Plant responses to abiotic stress conditions

Agricultural systems are intricately associated with climatic conditions and are highly susceptible to hostile and hazardous conditions. As per the projection, the evolving climate change could negatively impact the global agricultural production systems due to frequent extreme events (including drought, salinity, and thermal), thus affecting the quantity and quality of crop production tremendously (Buono, 2021). Currently, crops are more often subjected to biotic and abiotic stresses and thus causing losses of up to 50% in global agricultural production (Sangiorgio et al., 2020). BSt application is one of the sustainable methods for securing food security as it can enhance plant resilience against climate change–linked stresses (Calvo et al., 2014; Yakhin et al., 2017).

**Figure 1:**
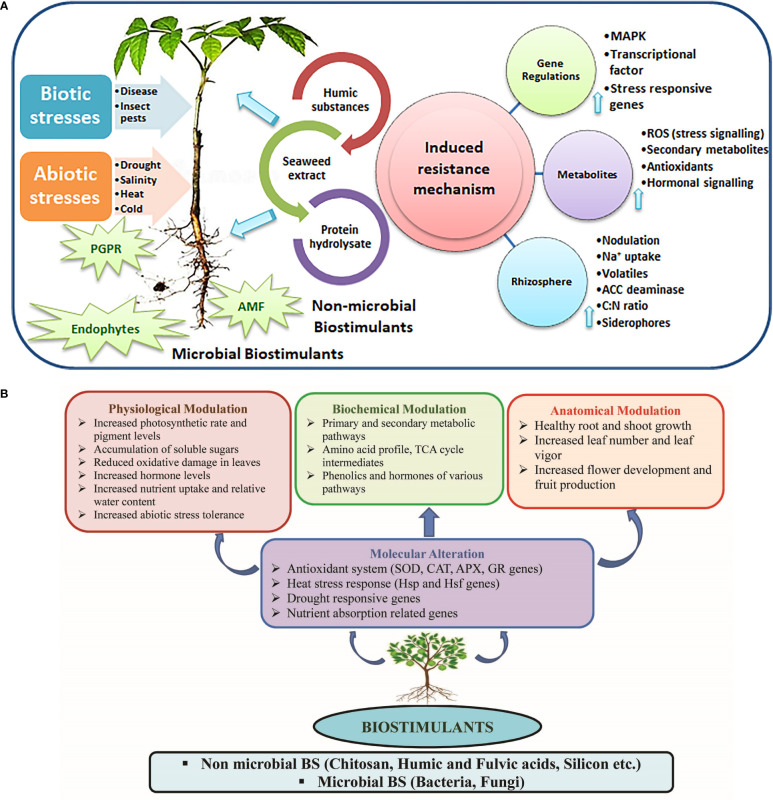
**(A, B)** The role of biostimulants in mitigating the adverse effects of stresses on plants through several mechanisms (molecular alteration, physiological, biochemical, and anatomical modulations).

The adverse effect of increasing temperature on agriculture may lead to injuries triggered by heat toward cells, disruption in protein synthesis, and functions of certain vital enzymes (Devi et al., 2017). Moreover, this could render a large agricultural area unfit for cropping over time due to high evapotranspiration and reduced soil moisture and quality, overpowering growth of various pests, and rising disease incidences (Prasad and Chakravorty, 2015). Drought conditions affect plants both physiologically and morphologically. These could exert harmful reactive oxygen species (ROS) accumulation (Smirnoff, 1993; Devi et al., 2017), ethylene release (Ali et al., 2014), and affect accessibility, assimilation, and translocation of plant nutrients (Rouphael et al., 2012; Devi et al., 2017). Further, salt accumulation in the soil under scattered and low–intensity rainfalls can aggravate the injury due to drought stress. Under high salinity levels, plants suffer from osmotic stress, negatively affecting their nutritional composition, metabolism, and growth (Bulgari et al., 2019). In the current scenario, soil salinization has impacted approximately 20% of the total cultivable areas (Shrivastava and Kumar, 2015). Moreover, drought stress and soil salinization are reportedly the main factors responsible for desertification, especially in over–exploited areas (Sangiorgio et al., 2020). In this context, BSts could play crucial roles in mitigating the negative effects of stresses on plants by inducing several mechanisms, including molecular alteration and physiological, biochemical, and anatomical modulations (**Figures 1A, B**). They also stimulate the innate immune responses of plants to biotic stress, in particular, by deploying cellular hypersensitivity, callose deposition, and lignin synthesis. Employing BSts like seaweed extracts, plant growth–promoting rhizobacteria, and humic acids augments the transcript of plant defense–related genes in crops (Agarwal et al., 2016; Pereira et al., 2021). BSts accelerate the antioxidative machinery to scavenge ROS overproduction to cope with adverse climatic conditions (Hasanuzzaman et al., 2021; Rakkammal et al., 2022).

## 3 Biostimulants: Overview and their types

Generally, biostimulants (BSts) are organic inputs applied to plants to enhance nutrition efficiency, abiotic stress tolerance, and crop quality traits, regardless of their nutrient content (du Jardin, 2015). Based on their origin, BSts are categorized into two broad groups: non–microbial BSts (chitosan, humic and fulvic acids, protein hydrolysates, phosphites, seaweed extracts, and silicon) and microbial BSts (arbuscular mycorrhizal fungi, plant growth–promoting rhizobacteria, and *Trichoderma* spp.) (Colla and Rouphael, 2015). Owing to their good solubility and multiplication ability in the rhizosphere, the application of BSts to the field and horticultural crops could overcome the yield barrier due to environmental stresses. Further, they can aid in realizing maximum potential yield with the minimum usage of synthetic fertilizers and pesticides.

BSts can be applied to crops through foliar fertilization, fertigation, or direct soil application, which enhances crop growth and development, and also crop quality (Van Oosten et al., 2017). Their vital functions are akin to fertilizers or by–products of plant protection which are an array of diverse microorganisms and components used to promote plant growth and development (Bhupenchandra et al., 2020). Recently, Lau et al. (2022) elaborated on the practical aspect of plant BSts and microbiome bioengineering on crop improvement and stress mitigation. BSt formulations can be directly applied to soil, alone or combined with micronutrients, as seed priming agents to anchor synergetic plant growth. BSts could sustainably reduce the environmental threats to plants, minimizing the negative consequences of indiscriminate chemical application.

### 3.1 Non–microbial biostimulants

#### 3.1.1 Humic substances

Humic Substances (HSs) are the products of aerobic microbial decomposition of organic materials broadly classified into Fulvic Acid (FA), Humic Acid (HA), and Humins based on their solubility at acidic and alkaline pH. HSs are a brown to dark–colored heterogeneous mixture of complex organic compounds such as sugars, polypeptides, fatty acids, and aliphatic and aromatic compounds interacting by hydrophobic bonds (Lipczynska Kochany, 2018; Shah et al., 2018; Guo et al., 2019). The formation, structure, classification, and physiochemical properties of HSs were described in detail by Sen et al. (2020). Oxygenated functional groups such as carboxylic and phenolic hydroxyl make HSs a reactive part of soil organic carbon (Conselvan et al., 2018). HSs can be extracted from various sources, including soil, vermicompost, earthworm cast, low–rank coals such as lignite or brown coal, leonardite, and peat coal, following different methods. Different extraction techniques were employed to extract HA from various sources, and their recovery efficiency was well–documented by Sarlaki et al. (2019) and Fatima et al., (2021).

HSs are one of the most commonly used BSts that are readily available and effective against various abiotic and biotic stresses. They enhance the growth and development of agricultural and horticultural crops by mitigating various environmental stresses by modulating the physicochemical characteristics of soil and the physiology of the plant. The physiological and biochemical effects of HSs on plant growth are highly influenced by their source and extraction methods (Huculak–Mączka et al., 2018). HSs trigger an array of physiological processes at different phases of plant growth (Canellas et al., 2015). They influence plant development by modulating functional proteins implicated in redox homeostasis, energy metabolism, protein synthesis, etc. (Roomi et al., 2018). They affect the primary metabolism in plant growth, evident from various studies showing induced upregulation of major genes implicated in nutrient acquisition, metabolism, and photosynthesis (Trevisan et al., 2011). In a nutshell, HSs boost soil organic carbon, which regulates the physio–biochemical events to gene expression associated with plant adaptation mechanisms and thus would be a game–changer in the sustainable agricultural production system.

#### 3.1.2 Seaweed extract

Seaweed Extract (SWE) is one of the most used BSts extracted from various seaweed species. Commercially available SWEs hold polysaccharides as the major components in which their fractions range between 30–40% (Battacharyya et al., 2015). Most of the commercially available SWEs are obtained from brown and red seaweeds. Brown seaweeds comprise *Ascophyllum nodosum, Fucus* spp., *Laminaria setchellii, Sargassum hildebrandtii, Turbinaria* spp., *Macrocystis pyrifera, Sargassum horridum, Ecklonia arborea, Durvillaea antarctica etc.*, and red seaweeds include *Kappaphycus alvarezii, Gracilaria edulis, Acanthophora spicifera, Gelidium robustum,* and *Gracilaria parvispora* (Singh et al., 2018; Sharma et al., 2019; Ali et al., 2021). Nature and nutrient composition vary among the extracts based on their source and extraction method (Mukherjee and Patel 2020). SWE possessed significant variations in oligosaccharides, polypeptides, phytohormones, terpenes, and polyphenols contents (Filippo–Herrera et al., 2019). SWE can alter the physiochemical nature of soil by altering the nutrient composition, enhancing the growth of beneficial microorganisms, and helping retain more water in the soil. In plants, its role varies from ameliorating nutrient stress to abiotic and biotic stresses by triggering various stress–responsive pathways, as it contains many secondary metabolites that participate in signal transduction. Upregulation of drought–responsive genes in soybean (Shukla et al., 2018), cold–response genes such as *COR15A, RD29A,* and *CBF3* in *Arabidopsis thaliana*, and downregulation of two chlorophyllase genes *AtCHL1* and *AtCHL2* have been reported in *Arabidopsis thaliana* (Stirk et al., 2020). The emerging trend of using SWE in the conditioning soil profile to augment beneficial microbes and plant nutrient amelioration to accelerate stress tolerance is well–proven in sustainable agricultural production (Abdullahi et al., 2021; Ali et al., 2021). Thus, wide application of SWE in the crop production system is highly recommended to induce plant metabolism to combat adverse environmental threats.

#### 3.1.3 Protein hydrolysate and N–containing compounds

Protein hydrolysate (PHs)–based biostimulants are a mixture of free amino acids, oligo– and polypeptides derived by chemical, enzymatic or chemical–enzymatic hydrolysis of plant residues or animal tissues (Caruso et al., 2019). PHs induce an array of defense responses under adverse plant growth conditions and pose growth and yield–promoting capability under non–stressed conditions. They improve leaf gas exchange through pigment synthesis and protection and improve water use efficiency (Colla et al., 2017; Rakkammal et al., 2022). In addition, PHs also exhibit hormone–like functions that modulate carbon and nitrogen metabolism and improve anti–oxidative properties under stress. PHs can be applied through seed priming, soil drenching, and foliar spray (Paul et al., 2019; Agliassa et al., 2021). PHs enhance plant growth and development (Colla et al., 2015) and alter root architecture by increasing root length, branching, and surface area (El–Nakhel et al., 2021). The Animal–based PHs at higher doses cause phytotoxicity and plant growth depression (Nardi et al., 2016). It is worth mentioning that exogenous PHs application positively influences the root health in the soil and equilibrates C: N ratio, which is considered the plant’s lifeline.

### 3.2 Microbial biostimulants

Microbial biostimulants (MBSts) promote growth and development in various crops. Certain microorganisms residing in the plant rhizosphere, particularly plant growth–promoting rhizobacteria (PGPR), act as natural growth–promoting agents (Backer et al., 2018; Del Carmen Orozco–Mosqueda, et al, 2020; Vasseur–Coronado et al., 2021). Soil–harboring microorganisms assist in the mobilization of micro and macronutrients to the soil rhizosphere (Vasseur–Coronado et al., 2021). Various MBSts are available as ready–to–use commercial formulations are enlisted in **Supplementary Table 1**. These microbial inoculants play a crucial role in nutrient acquisition through solubilization of phosphate, siderophore synthesis, N–fixation, and disease suppression. MBSts are involved in various signaling cascades through the nature of phytohormones, secondary metabolites, amino acids, polysaccharides, and antibiotics (Gupta et al., 2021). Numerous arbuscular mycorrhizal fungi (AMF) and *Trichoderma* spp. represent important categories of MBSts. Moreover, various soil microorganisms, including *Azospirillum* spp., *Bacillus* spp., *Arbuscular mycorrhizae, Pseudomonas* spp., *Paraburkholderia phytofirmans*, and *Promicromonospora* spp., were identified as potent BSts and respond to various abiotic stresses (Kang et al., 2012; Duc et al., 2018; Esmaeel et al., 2018; Crovadore et al., 2020; Del Carmen Orozco–Mosqueda et al., 2020; Oliveira et al., 2020). Endophytic MBSt, the micro guest in plants, often confront the invaders by releasing secondary metabolites that scavenge reactive oxygen species owing to stress. Thus, MBSts could be isolated, characterized, and promoted to enhance biotic and abiotic stress tolerance in different crops.

## 4 Role of biostimulants in plant growth promotion

### 4.1 Nutrient acquisition and mobilization

BSts are well known for their role in nutrient uptake and mobilization (**Supplementary Table 2**). Carbon–rich BSts such as HSs are rich nutrient reservoirs. FA enhances the ability of the plant to acquire more nitrogen (NO_3_) through increased nodulation, improved protein activities involved in NO_3_ uptake and assimilation, and even modifies at the transcriptional level (Moshe et al., 2015; Capstaff et al., 2020). Dissolved HSs alter the nitrogen metabolism through upregulation of genes implicated in the nitrate acquisition, such as *ZmNRT2.1, ZmNRT2.2* coding for two high–affinity nitrate transporters and *ZmMHA2* for a plasma membrane H^+^–proton pump as well as *ZmNADH:* NR, *ZmNADPH: NR*, and *ZmNiR* which are involved in nitrate and nitrite reduction, respectively (Vujinović et al., 2020). GS and GOGAT are the key enzymes involved in nitrogen metabolism and are HS derived from leonardite (Ertani et al., 2009; Conselvan et al., 2017). Noroozisharaf and Kaviani (2018) found that the application of humic acid significantly increased nutrient uptake (N, P, K, Mg, and Fe) in *Thymus vulgaris*. GomesJúnior et al. (2019) demonstrated the effect of HA derived from vermicompost on different nutrient acquisition and metabolism, and they inferred that HA influenced the membrane fluidity, the activity of the pyrophosphatase enzyme (H^+^–PPase), augmented the activation of the plasma membrane H^+^–ATPase enzyme which further facilitated in N, P and K uptake and amplified the uptake of other essential plant nutrients such as S, Ca, Mg, Mn, and Fe. Genes encoding Fe (III) chelate–reductase *(CsFRO1)*, H^+^–ATPase (*CsHa1* and *CsHa2*), and Fe (II) transporter *(CsIRT1)* are transcribed in higher amounts under the influence of leonardite derived HA (30–80%) which ultimately resulted in higher Fe accumulation in aerial parts of the plants (Aguirre et al., 2009). Even under the alkali soil stress condition, the application of HS could enhance the assimilation of micronutrients (Chen et al., 2004; Sanchez–Sanchez et al., 2006). However, plant nutrient acquisition is prominently affected by several factors, including the concentration of HS applied and the nature of soil’s physical and chemical properties. HSs such as HA and FA improve the soil structure and help the solubility of soil micronutrients. The formation of clay–humic complexes due to HS application improves soil aeration, minimizes soil erosion, facilitates more root penetration, and better water availability to the plant (Bronick and Lal, 2005). HSs form a metal–humic complex that enhance the micronutrient solubility in soil water (Chen et al., 2004). In plants, they activate H^+^ATPase and NO_3–_ assimilating enzymes and modify the root morphology by increasing root surface area and lateral and root hairs (Halpern et al., 2015). Therefore, HSs are involved in better nutrient assimilation by altering soil properties and plant responses. A significantly higher diffusion coefficient (mobilization) percentage (96%) coupled with Fe and FA application to the calciorthent soils. The soil organic content was positively correlated with the diffusion coefficient of Fe (Pandeya et al., 1998). An increase in Fe diffusion could be attributed to the decrease in the ‘capacity factor’ of Fe (Gupta and Deb, 1985).

The nutrient deficiency conditions created by low–temperature stress can be overcome by applying SWE (Van Oosten et al., 2017). Micro and macronutrients, along with amino acids, vitamins, and phytohormones– substances (auxins, cytokinins, ABA), are abundantly present in SWE, which can determinately change the cellular metabolism and ultimately impact plant growth and yield (Khan et al., 2009) and plant chemical composition (Tursun, 2022). Moreover, there was a positive correlation between the SWE dose applied as a foliar and the nutrient content in soybean grains (Rathore et al., 2009). There is evidence of the synergistic relationship of various chemical components of SWE with crop productivity via upregulation and downregulation of various genes coding plant growth, regulation of phytohormones signaling, primary metabolism, and antioxidant activity (Kumar et al., 2020; Trivedi et al., 2021). SWE is used in low concentrations (1:1000 or more), which could have facilitated its easy penetration inside the plant tissue (Crouch and van Staden, 1993). Being carbon–rich material, HSs act as a food source for various plant growth–promoting microorganisms (PGPM) in the rhizosphere. The symbiotic relationship between PGPM with legumes (Vessey, 2003) and other crops (Sanginga, 2003) has enhanced nitrogen assimilation. Also, PGPM has been noted to improve the solubility of P (Vessey, 2003), Fe (Sharma et al., 2003) and modify the root morphology for better nutrient transport in plants (Lopez–Bucio et al., 2007; Halpern et al., 2015) and improve physicochemical properties of soil (Sughra et al., 2021). PGPM (*Bacillus, Brevibacillus,* and *Rhizobium* spp.) increases chlorophyll content by reducing the degradation rate or enhancing the pigments synthesis rate, thereby increasing the rate of photosynthetic and ultimately augmenting the crop yield (Khan et al., 2009). Furthermore, *Pseudomonas* sp. enhanced the activities of antioxidants which could aid in better managing drought stress conditions (Heidari and Golpayegani, 2012). Microalgae–cyanobacteria–based BSt improved the overall nutrient content by increasing the root biomass, enhancing K^+^ uptake, and reducing Na ^+^/K^+^, thereby helping maintain the ion homeostasis even under salinity stress (Mutale Joan et al., 2021). The microbial–based BSts containing AMF and *Trichoderma koningii,* regardless of water regimes, enhanced the uptake of P, Mg, Fe, Mn, and Zn by many folds, the content of various phenolic compounds, and plant yield (Saia et al., 2019). Thus, BSts can be used as a potential agent to improve the overall nutrient status of crop plants. The practical use of BSts in various crop production has been summarized in **Supplementary Table 2**.

### 4.2 Implications of biostimulants for abiotic stresses tolerance

#### 4.2.1 Drought stress tolerance

Drought is the principal abiotic stress that affects plant performance and quality. It has been reported that applying microbial and non–microbial–based BSts significantly impacts plant growth and development, micro–and macronutrient uptake, and translocation in several crops, ultimately leading to increased biomass production and yield (Rouphael and Colla, 2020). At the same time, BSt application also induces tolerance to several biotic and abiotic stresses in agricultural and horticultural crops. For instance, pollen grain extract (PGE) @ 1 g/L as a foliar application improved relative water content and water use efficiency, lowered electrolyte leakage, enhanced plant growth, antioxidant enzyme activities, and essential oil productivity of *Ocimum basilicum* under drought conditions (Taha et al., 2020). BSts like seaweed extracts, humic substances, amino acids, protein hydrolysates, and several beneficial microorganisms on vegetables counteract the most common abiotic stresses, including drought stress (Bulgari et al., 2019).

Pre–treatment with brown alga *Ascophyllum nodosum* before drought conditions in *Arabidopsis* enhanced the water use efficiency and mesophyll conductance, assisted in retaining a robust stomatal control, induced a part stomatal closure through a lesser expression of MYB60, and even led to alterations in the expression levels of genes associated with ABA–responsive and antioxidant system pathways (Santaniello et al., 2017). The researchers also pointed out that the expression of genes like *NCED3, RAB18,* and *RD29A* increased sharply during the last phase of the dehydration period, when a steep decline of stomatal conductance was recorded, which suggested that a robust synthesis of ABA was undergoing close stomata and counteract the water loss. Excellent results have also been obtained from the use of SWE on broccoli and spinach (Lola–Luz et al., 2014; Franzoni et al., 2022), canola (Shahriari et al., 2021), and grapevine (Irani et al., 2021) under drought stress. Besides the direct and indirect effects of HA on plant growth, metabolism, and physiological pathways, many studies have reported their bio–stimulatory activity in stress resilience, especially against drought and salinity (Garcia et al., 2012; Petrozza et al., 2014). The mechanisms of salt and drought involve (a) reduced level of hydrogen peroxide and lipid peroxidation, (b) enhanced proline content, (c) differential regulation of gene expression under stress conditions, (d) improving the soil physicochemical and biological attributes, and (e) enhance the root growth of the plants (Calvo et al., 2014; Battacharyya et al., 2015).

Pure organic compounds can be used as natural BSts to combat various stresses faced by plants (Garcia–Garcia et al., 2020). Glutamate elevated resilience to drought in canola by enhancing the synthesis of proline biosynthesis genes and the concentration of compatible osmolytes (La et al., 2020). In water–deficit maize (*Zea mays* L.), a foliar application with proline improved the overall plant growth and mitigated the adverse impacts of drought conditions (Ali et al., 2008). Proline, as an osmoprotectant, executes drought stress tolerance in barley (Abdelaal et al., 2020) and tobacco (Hoque et al., 2007). Foliar sprays with proline up–regulated the activities of antioxidant enzymes, thereby increasing resilience to drought and high–temperature environments (Hanif et al., 2020). Glycine–betaine (GB) is another osmolyte other than proline. Foliar and root application with GB compensated the side effects of salinity and drought stress in different crops like corn (*Zea mays*) (Ali and Ashraf, 2011), kidney bean (*Phaseolus vulgaris* L.) (Sofy et al., 2020), broad bean (*Vigna faba* L.) (Gadallah, 1999), barley (*Hordeum vulgare* L.) (Wang et al., 2019), and lettuce (*Lactuca sativa* (Shams et al., 2016). Also, the treatment of Gamma–Aminobutyric acid (GABA) improved stress tolerance in the plant (Bown and Shelp, 2016) by improving the osmoregulation, production of energy, and secondary metabolites in Agrostis stolonifera (Li et al., 2017). The production of GABA in *Arabidopsis thaliana* under stressful environments stimulates drought tolerance in *Vicia faba* through upregulation of stress–related gene transcription. It is also reported that GABA–induced drought tolerance in maize through the jasmonic acid pathway by activating antioxidant defense mechanisms and abscisic acid synthesis (Shaw et al., 2016). Also, chitosan stimulates drought tolerance in crops like maize (Rabêlo et al., 2019) and barley (Hafez et al., 2020). This treatment augmented antioxidant mechanisms, photosynthesis, and grain yield. Zeng and Luo (2012) reported that coating wheat seedlings with chitosan imparted drought tolerance by increasing antioxidant production and chlorophyll content.

Alginate oligosaccharides (AOS) are polymers that are extracted from marine brown algae and are reported to enhance tolerance to PEG–induced drought stress in wheat (Liu et al., 2013), tomato (Liu et al., 2009) and cucumber (Li et al., 2018) through the expression of drought resistance genes and regulated ABA–dependent signal transduction. Xu et al. (2020) identified alpha–PGA as a drought–mitigating agent that can lessen to adverse impacts of drought on plants. The external application of synthetic organic molecules like acetic acid is known to improve drought tolerance in *Arabidopsis,* rice, maize, wheat, and rapeseed through the promotion of jasmonic acid synthesis and enrichment of histone H4 acetylation (Kim et al., 2017). This compound has been observed to encourage abscisic acid build–up in rape (*Brassica napus* L.) and augment the enzymatic antioxidant system and accumulation of proline.

Natural and artificial sources of vitamin C effectively alleviate the adverse impacts of drought in various crops (Aziz et al., 2018), including *Chenopodium quinoa, Phaseolus vulgaris* (Gaafar et al., 2020), and *Vigna faba* (Desoky et al., 2020). Similarly, vitamin E in Chinese ryegrass (*Leymus chinensi*) seedlings (Gu et al., 2008) and wheat (Ali et al., 2019) improved drought resilience by augmenting antioxidant defense systems, WUE, photosynthetic pigments content, and photosynthetic efficiency. Application of BACSTIMR 100 (Omnia Group Ltd., Bryanston, South Africa), a MBSt formulation of five *Bacilli* strains, conferred enhanced resilience to drought in maize plants through alteration of vital metabolic pathways involved in drought tolerance mechanisms like the redox homeostasis, osmoregulation, enhanced energy production, strengthening of the cell wall and membrane remodeling (Nephali et al., 2021). Exogenous applications of cis– and trans–zeatin, cytokinins, and plant extracts are known to enhance the tolerance of plants to salt and drought stress (Schafer et al., 2015). Alharby et al. (2020) found that seed pre–treatment with zeatin or maize grain–derived organic BSt upgraded the hormonal contents, polyamine gene expression, and abiotic stress tolerance like salinity and drought in wheat.

The molecular insights of BSt–anchored drought tolerance mechanisms open up a new platform for sustainable agriculture. Recent molecular studies unravel the pathways triggered by specific BSt at the cellular and gene levels (Baltazar et al., 2021). Different techniques like *in vitro* bioassays, high–throughput phenotyping, micro–phenotyping, multi–trait high–throughput screening (Smith et al., 2021), microarray techniques (Geelen and Xu, 2020), sequencing techniques like Illumina IG, and very recently, *omic* approaches *i.e.*, transcriptomics, proteomics, and metabolomics have clarified the molecular basis of the phenological changes induced by the application of BSts (Xu et al., 2020). Several molecular mechanisms coordinate the plant defense responses against abiotic stresses, fine–tuning changes inside the plant system (Lephatsi et al., 2021), and alterations in the plant transcriptome, proteome, and metabolome (Fernandes et al., 2008; Li et al., 2008). Various adaptive responses are activated, and stress–related genes regulated by transcription factors (TFs) are expressed upon the perception of stress signals. A single TF regulating the expression of numerous genes through the specific binding thereof to the cis–and trans–element in the promoters of target genes is called regulon (Nakashima et al., 2009). These regulons, activated in response to abiotic stresses like drought, are components of ABA, a major phytohormone that regulates an intricate gene regulatory system that makes plants tolerant to several environmental perturbations. In a recent study, a new BSt (EnNuVi® ALPAN®) was assessed for its efficacy on tomato plants subjected to drought environments and the molecular effects and gene expression were illuminated (Hamedeh et al., 2022). ALPAN® upregulated the gene expression involved in translocation and source to sink carbohydrate metabolism, stomatal closure, and cell homeostasis. In maize, an alteration in gene expression was observed in the presence of HA, particularly in the genes for nitrate transporters (*Nrt2.1* and *Nrt1.1*) (de Azevedo et al., 2019), PM–H^+^–ATPase (*Mh1*), a gene fundamental in the electrochemical gradient of cell membranes leading to better absorption of nutrients (Morsomme and Boutry, 2000) and aquaporin 1 (*PIP1*) (Singh et al., 2020), which aids in water and solutes movement. An alteration in gene expression due to the application of HA during drought stress was reported in wheat (Arslan et al., 2021). Park et al. (2009) used chemical genomics approaches to discover the ABA receptor family proteins and to unravel fundamental biological questions related to drought stress. Okamoto et al. (2013) isolated compounds capable of activating different ABA receptors, *PYR1, PYL1, PYL2, PYL3,* and *PYL4*, using yeast two–hybrid strains to identify stress–protective small molecules. The reporter genes that play crucial roles in abiotic stress responses recognize these small molecules and elicit the accumulation of transcripts that ideally trigger downstream responses resulting in enhanced stress tolerance. For instance, *Arabidopsis* plants overexpressing *UGT74E2* showed enhanced tolerance to drought and salt stresses by counteracting the adverse effects of abiotic stress. A 1500–bp region upstream of the *UGT74E2* start codon was fused to a luciferase reporter to identify small molecules capable of inducing *UGT74E2* (Kerchev et al., 2014). Summarily, BSts induce stress tolerance in two approaches: first, developing the avoidance mechanism involving morpho–physiological adaptation, and second, tolerance mechanism involving biochemical, biomolecular, and gene regulations in the plant.

Recent advancement in various *omics* techniques has made it achievable to exemplify an organism’s molecular profile in a high–throughput and inclusive manner (Benevenuto et al., 2022). Seaweed extract–based BSts are a potential and sustainable approach for improving crop growth and stress resilience (Tinte et al., 2022). To know the insights of cellular, biochemical, and molecular mechanisms governing the benefits of the seaweed extracts on plants, a liquid chromatography–mass spectrometry–based untargeted metabolomics approach combined with computational metabolomics strategies was used to unravel the molecular mechanism of SWEs (Kelpak® formulation, a registered biostimulant made from kelp *Ecklonia maxima*) on greenhouse–grown maize (*Zea mays*) under drought stress. It was observed that applying SWEs alters various pathways of primary and secondary metabolism, like flavonoid and phenylpropanoid biosynthesis, fatty acid metabolism, and amino acids pathways. These altered pathways further enhance acid metabolites such as tryptophan, phenylalanine, linolenic, and coumaroylquinic acid, which are related to physiological and biochemical events that enhance drought resistance traits. In several studies, *Kappaphycus alvarezii* SWE has been reported to enhance yield and drought tolerance in maize (Trivedi et al., 2018, 2022; Kumar et al., 2020). Kumar et al. (2020) sequenced mRNA using a high–throughput Illumina platform, and transcriptome mapping was carried out. *Kappaphycus alvarezii* SWE applied to maize plants under drought, when compared to its control, recorded an up–regulation in the genes coding for enhancement of root growth and seed development; signaling of gibberellic acid and auxin; nitrogen metabolism and transport; and antioxidant activity like glutathione S–transferase and peroxidases. Layek et al. (2018) assessed the performance of SWEs viz. *Kappaphycus alvarezii* and *Gracilaria edulis* in ameliorating the productivity and quality of rice by applying 100% recommended dose of fertilizers and showed that high concentrations (10% and 15%) of both extracts improved significantly the N and P uptake, but not K. Sharma et al. (2019) also reported a significant increase in wheat biomass and yield under drought stress when *Gracilaria dura* extract was applied to plants. The *Gracilaria* extract facilitates the mechanisms involved in water–saving strategies, thereby rendering the wheat plants tolerant to drought.

#### 4.2.2 Salinity stress tolerance

Salt stress is one of the chief abiotic stresses that limit agricultural production and global food security and environmental sustainability in recent times (Mukhopadhyay et al., 2021; Ondrasek et al., 2022). It occurs due to high temperature–induced evaporation, decreased precipitation associated with degraded water quality, and salt–contaminated groundwater (Akbari et al., 2020). About 20% of yield losses occur due to salinity stress worldwide (Rehman et al., 2005). The presence of excess salt in the soil in soluble form changes the plant’s normal physiological processes and affects plant growth explaining the salt stress. **Supplementary Table 3** summarizes the adverse effect of salinity stress on crop growth and development. In general, 20% of the total cultivated and 33% of the irrigated agricultural lands are affected by salt stress (Mukhopadhyay et al., 2021). Mainly salinity stress is observed in arid and semi–arid regions and coastal areas where coastal flooding occurs due to a rise in sea level, which leads to salt invasion into the soil, severely affecting the soil quality (Shrivastava and Kumar 2015).

The application of non–toxic organic and eco–friendly methods such as BSts could improve crop growth, development, and stress tolerance, such as salt stress resilience (Ondrasek et al., 2022). BSts alleviate the salinity stress in different plant species through modification of physiological processes and consequently optimize productivity and growth in the plant. BSts of different origins improve the plant’s resistance to salinity conditions by upregulating the genes responsible for stress tolerance (Campobenedetto et al., 2021; Islam et al., 2021). Tognetti et al. (2010) opined that the *UGT74E2* gene could play a conserved role in specific stress environments, including high salinity conditions, by upregulating the accumulation of UDP–glucosyltransferase with a glycosylation activity and thus enhancing the biosynthesis of indole butyric acid. BSt is one of the novel potential approaches for modifying physiological processes in diverse species of plants.

To survive in high salt concentrations in soil, plants develop several modifications of biochemical and physiological mechanisms that were observed, and their detail is shown in **Supplementary Table 4**. **Supplementary Table 5** depicts mitigation approaches of various BSts to salt stress. Bandopadhyay et al. (2021) evaluated the salt tolerance level of *Brassica juncea* b–85 through physiological (germination, seedling growth) and biochemical analysis (Na ^+^and K^+^, chlorophyll, total soluble sugar, and glycine betaine content). The result revealed a high strength of 200 mM NaCl as the lethal dose of 50 for B–85 germination and seedling growth. The salt–induced increase in Na^+^/K^+^ ratio was higher in roots than shoots suggesting the presence of active regulation to protect the sensitive photosynthetic structures, which indeed manifested protection well up to 200 mM of NaCl stress, as total chlorophyll content showed significant reduction only at 300 mM NaCl stress level. At 200 mM of NaCl stress, glycine betaine has a potential role in maintaining Na^+^ and K^+^ homeostasis, photosynthesis, and overall growth of the *B. juncea* B–85 variety. Different BSts used to mitigate salt stress are listed in **Supplementary Table 5**. We found the importance of different BSts under salinity stress in diverse crop species to improve the crop yield and amend the salinity stress. It also has the prospective to develop plant resilience to adverse climatic conditions and gives a better yield to crop plants. From the above study, we can mention that the enhancement of salt tolerance variety can be developed through selection and breeding methods. Selecting genotypes with the maintenance of high tissue K^+^/Na ^+^and Ca2^+^/Na ^+^ ratios is an important selection criterion for salt tolerance in brassica Species (Ashraf and McNeilly, 2004). External application of Glycine betaine and transgenic plant accumulating Glycine betaine showed reduced Na^+^ concentration in leaf and increased K^+^ content. A high K^+^ and low Na+ concentration is very important in a plant cell to carry out its physiological activities (Wei et al., 2017).

#### 4.2.3 High–temperature stress tolerance

The increasing temperature causes an alteration in the period of growth, the distribution of crop plants, and the crop yield level. According to the report of Peng et al. (2004), there was a yield decline of about 15% per 1°C rise in rice between 1979 and 2003. These could be attributed to the alteration in physiological and biochemical processes in plants under elevated temperatures. High temperature has been reported to damage the membrane, some proteins, inactivate main enzymes, disturb the synthesis of biomolecules, and restrict the process of cell division. Elevated temperatures also affect the physiology of the plant by increasing respiration and transpiration rates and altering photosynthate allocation (Munns, 2002; Malhotra, 2017). Rubisco’s affinity for carbon dioxide decreases at high temperatures; however, its affinity for oxygen increases (Sangiorgio et al., 2020). With the increase in temperature, the solubility of carbon dioxide decreases compared to oxygen, hence lowering the concentration of carbon dioxide in the chloroplast (Ku and Edwards, 1977). Furthermore, plants shut their stomata when the temperature increases to prevent or decrease evapotranspiration loss. Carbon dioxide concentration declines rapidly with the closure of stomata while the concentration of oxygen increases which limits photosynthesis and increases photorespiration (Bhattacharya, 2019). Heat stress results in changes in the molecular, biochemical, and physiological reactions of the plants (Sangiorgio et al., 2020), which results in the expression of heat shock proteins (HSPs). These enzymes degrade ROS, osmoprotectant chemicals, amino acids, sugars, and sulfur compounds (Shulaev et al., 2008) as a plant defense mechanism (Qu et al., 2013).

Higher temperatures during the spring season make annual crops grow many cycles in a year, such as tomato (Sangiorgio et al., 2020) and lettuce (Pearson et al., 1997) and resulting in early flowering in many horticultural crops (Wheeler et al., 1993; Bisbis et al., 2018). However, it promotes male flower development and suppresses female flower development in cucumber (Sangiorgio et al., 2020), which affects the total yield. In temperate fruit crops like apple (Funes et al., 2016), peach, and plum (Hazarika, 2013), which require chilling temperature for flower differentiation, hampers crop production due to high temperature (Luedeling, 2012). BSts, including MBSt, could render an important stimulatory role in mitigating plant responses to heat stress (**Supplementary Table 6**). The synthesis of ROS–degrading enzymes could enhance the heat stress tolerance in plants. The same benefit was observed in plants colonized by microorganisms like *Pseudomonas, Bacillus, Septoglomus deserticola, and Septoglomus constrictum* (Duc et al., 2018). SoilPro® (Liventia, TX, United States) is a MBSt containing *P. fluorescens* and *P. aeruginosa* and helps improve soil quality and heat stress tolerance (Sangiorgio et al., 2020). Hormonal signaling is in charge of heat stress responses, and ethylene is involved in senescence, development, plant physiology, and development and heat stresses (Byerlee et al., 2014; Dubois et al., 2018). The harmful effect of heat stress can be reduced by using microorganisms that limit ethylene production (Glick, 2014). Bacteria such as *Bacillus subtilis* BERA 7, *Leclercia adecarboxylata* MO1, *Pseudomonas fluorescens* YsS6, and *Pseudomonas migulae* 8R6 have exhibited 1–ACC deaminase activity (Del Carmen Orozco–Mosqueda et al., 2020). *Paraburkholderia phytofirmans* strain PsJN which expresses ACC deaminase activity to alleviate and induce tolerance toward heat stress, has been reported in tomato (Esmaeel et al., 2018) and potato (Bensalim et al., 1998). The better performance of the plant growth under heat stress with the use of PsJN is due to the development of more secondary roots, which leads to more water and nutrient availability and its ability to accumulate cytokinin and increase pH in the treated plants. Though this bacterium has promising beneficial activity, its commercial use is still lacking.

Khan et al. (2020a) studied the effect of thermos–tolerant SA1, an isolate of Bacillus cereus and HA, on tomato seedlings. They observed that the combined application of SA1+HA significantly improved the biomass and chlorophyll fluorescence of tomato plants under heat–stress conditions. It also reduced abscisic acid and decreased salicylic acid content. The plants treated with a combination of SA1 and HA resulted in increased activity of antioxidant enzymes such as ascorbate peroxidase (APX), superoxide dismutase (SOD), and reduced glutathione (GSH). Inductively–coupled plasma mass spectrometry showed significantly higher Fe, P, and K uptake during heat stress. Heat stress increased the relative expression of *SlWRKY33b* and autophagy–related (*SlATG5*) genes, while the application of SA1+HA in combination augmented the heat stress response and reduced *SlWRKY33b* and *SlATG5* expression. The heat stress–responsive TF (*SlHsfA1a*) and high–affinity potassium transporter (*SlHKT1*) was upregulated in SA1+HA treated plants. Applying SA1+HA in combination can be used to mitigate heat stress in tomatoes. Similarly, Khan et al. (2020b) in soybean identified thermos–tolerant *B. cereus* SA1 that could produce biologically active metabolites such as GA, IAA, and organic acids. Soybean plants inoculated with SA1 showed improved biomass, chlorophyll content, and chlorophyll fluorescence under normal and heat stress conditions for 5 and 10 days. The increase in chlorophyll content may be due to increased photosynthetic leaf area, enhanced moisture retention, and improved nutrient supply in the root zone. It was also observed that after five days of heat stress, HSP expression increased, while *GmHSP* expression was reduced after ten days. However, SA1 inoculation augmented the heat stress response and increased HSP expression. Plants inoculated with SA1 overexpressed the stress–responsive *GmLAX3* and *GmAKT2,* which may be associated with reduced ROS generation, altered auxin and ABA stimuli, and enhanced potassium gradients, which are critical in plants under heat stress.

Dudas et al. (2016) observed that lettuce (*Lactuca sativa* L.) production on the Adriatic coast was hampered by heat and water shortages in summer and the deficiency of light and frost in winter. They found that the use of BSt Bio–algeen S–90, derived from the brown seaweed *Ascophyllum nodosum* (L.) Le Jolis, had a better effect on plant height, leaf number, head mass, vitamin C, and dry matter content in lettuce leaves. It could be due to the synthesis, transport, and accumulation of auxins in lettuce. High temperature causes physiological stress in bell pepper grown in the greenhouse. However, bell pepper cultivars treated with BSts (Radifarm®, Megafol®, Viva®, and Benefit®) showed a notable increase in yield (Parađikovic et al., 2013). *Ascophyllum nodosum*–derived SWE induced the upregulation of heat stress–associated genes related to different transcription factors (TFs) and HSP families. *AtHSP17,* a family of HSP (particularly *AtHPS17.4* and *AtHPS17.6A*), is strongly expressed under the BSt treatment (Cocetta et al., 2022). Significant upregulation of *HSP101.1* and *HSP70.9* in flowers and fruits is reported to play a crucial role in temperature tolerance in tomatoes when supplied with the carbohydrate–rich fraction of A. nodosum. (Carmody et al., 2020). Thus, BSts enable plants to tolerate high temperatures by reducing photorespiration, antioxidant enzyme upregulation of HSPs, and participating in the stress signal transduction and stress response.

#### 4.2.4 Low–temperature stress tolerance

Crops, whether vegetables, fruit trees, woody crops, or cereals, grow and develop within an optimal range of maximum and minimum temperatures. Every crop species has a specific capacity to tolerate stress at low temperatures. Extreme cold and sudden temperature drops can be lethal to the crops due to the formation of tiny ice crystals on the outside and inside plant cells. This could restrict the plant’s metabolic processes and hamper photosynthesis, respiration, or the translocation of essential nutrients and water, ultimately affecting the plants’ growth and development, and yield. Generally, the stress tolerance capacity in crops is determined based on the cultivar’s ability to withstand freezing temperatures; however, with advancements in microbiological techniques, several stress–tolerant microbes have been identified which alleviate temperature stresses in plants. MBSts can help to reduce the effect of chilling temperatures in plants by producing growth–related hormones or that ethylene concentration (Sangiorgio et al., 2020). Applying soil with psychrotolerant (cold tolerant) bacteria can offer chilling tolerance. The psychrotolerant soil bacterium *Burkholderia phytofirman* is a PGPR capable of colonizing multiple plant species. Van Oosten et al. (2017) found that *B. phytofirman* augmented the chilling tolerance in grapes through effective ROS scavenging metabolism and upregulation of stress–induced genes. Inoculated plants recovered faster from chilling stress, returning to normal metabolic levels more quickly than untreated counterparts.

Wheat seedlings inoculated with bacteria such as *Pantoea dispersa* 1A and *Serratia marcescens* SRM strains which produce IAA, exhibited considerably better nutrient and yield absorption ability than control when cultivated under cold conditions (Selvakumar et al., 2008; Amara et al., 2015). Likewise, increased cold tolerance was observed in wheat seedlings when treated with *Pseudomonas* sp. NARs9 and PGERs (Mishra et al., 2008 and 2012). Wang and Shaohua (2006) reported that pre–treatment of exogenous salicylic acid could induce heat or cold tolerance in grapes by decreasing thiobarbituric acid–reactive substances and relative electrolyte leakage under heat or cold stress. Enzymes such as APX, glutathione reductase, monodehydroascorbate, and redox ratio in the ascorbate–glutathione were relatively higher in the grape leaves under average temperature and heat or cold stress. Gavelienė et al. (2018) studied the effect of BSts (Ruter AA, Terra Sorb, and Razormin) containing free amino acid and macro–and microelements on freezing resistance in winter rapeseed and winter wheat under controlled cold conditions by applying morphometrical methods. They found that the BSts applied under controlled cold stress conditions and natural conditions increased the freezing tolerance of winter wheat and rapeseed seedlings. In a study, Tiryaki et al. (2019) observed that beans inoculated with psychrophilic ACC deaminase–producing bacteria (*Psuedomonas fragi, P. fluorescens, P. proteolytica, B. frigoritolerans,* and *P. chlororaphis*) had reduced ROS production and lipid peroxidation, which could impart better frost resilience in them. MBSts are found as biopesticides (Cedomon®, BioAgri AB, Uppsala, Sweden) in the market, while others are formulated in combination with similar PGPR products. The bean plants were grown under the controlled condition at sub–optimal temperatures (<200C) and were treated with FH Attivus® biostimulant. The result showed that the beans were more tolerant to low temperatures by maintaining CO_2_ net assimilation rate A and increased antioxidant enzyme activity. The relative chlorophyll content (SPAD index), PSII effective quantum yield of linear electron flux, apparent electron transport rate (ETR), and photochemical extinction coefficient were higher in all treatments compared to the control (Do Rosário Rosa et al., 2021). Eggplant is a warm–climate crop, and unfavorable growing conditions like low temperature affects its flowering and fruit set. Pohl et al. (2019) investigated the flowering biology of three eggplant hybrids treated with biostimulant SWE Göemar BM–86® (Arysta LifeScience North America, LLC) to determine its yield in temperate climatic conditions. These BSts increased the percentage of medium and long–style flowers, ultimately improving the reproductive effectiveness under low–temperature stress.

**Supplementary Table 6** summarizes various BSts, their application mode, and the tolerance mechanisms responsible for mitigating high and low–temperature stresses. The application of BSts could be a remarkable effort to combat climatic vulnerabilities such as drought, salinity, temperature stresses, etc. BSts alter plant growth behavior through physiological, biochemical, and anatomical modulation of plants (as depicted in **Figure 1B**). Although numerous BSts promote plant growth under unfavorable conditions, limited BSt products are available for addressing the problems emphasized by climate change. The high cost for the production of commercial BSt, the complex registration process for a commercial product, and the variability in its efficacy in field conditions are some of the major hindrances in developing BSt products on a commercial scale. To develop or select a potential MBSt, in–depth characterization of plant microbiomes (especially native ones) through the application of next–generation sequencing technologies, real–time monitoring of the dynamics of microbial functions, and developing, optimizing, and understanding plant–microbe ecological interactions are needed.

Many researchers have already necessitated bridging the knowledge gap regarding the functional mechanism of BSts, such as their mode of action, application rate, and biostimulant–plant specificities, via a combined approach utilizing biology, chemistry, and omics. These techniques approach provides information on the mechanisms of BSt by identifying the biochemical and molecular pathways affected as well as proposing their regulatory role in the pathway (Campobenedetto, et al., 2020; Gonzalez–Morales et al., 2021; Ali et al., 2022; Brazales–Cevallos et al., 2022; Della Lucia et al., 2022;). The development of new BSt products should include certain microorganisms with different PGP functions so that it targets multiple stresses in one go rather than a single specific target. This heterogeneous nature of bio–stimulants may evade legislative categories of pesticides, fertilizers, etc., and elude long and expensive trial procedures before approval and commercial release.

## 5 Modern approaches and perspectives to study the role of plant biostimulants

To develop and fully implement BSts for sustainable agriculture and alleviation of the impact of climate change, a comprehensive analysis of crop yields in the open–field trials with BSt application under different parameters could provide us an idea about the optimum conditions and also help in the development of effective BSts following the guidelines of the Fertilizing Products Regulation (Ricci et al., 2019). Molecular studies involving studies at genome, mRNA, protein, and metabolite levels provide a clear picture of the impact of BSts on the plant under study. Through genome sequencing, we can discover changes or mutations in gene sequences and their regulatory elements that may arise due to stress responses. Vaghela et al. (2022) attempted to characterize and execute metabolomics profiling of *Kappaphycus alvarezii* SWE and comprehend its linkage with recognized bioactivities. From the above study, kinetin, dodecanamide, 1–phosphatidyl–1D–myoinositol, sulfabenzamide, and several other compounds have prominent bioactivities like plant growth promotion, photo–protection, hormone signaling, disease resistance, anti–microbial, antioxidant and anti–herbivory properties. Crovadore et al. (2020) reported a draft genome sequence of Bacillus licheniformis Strain UASWS1606, a potential BSt for agriculture. *Bacillus licheniformis* strain UASWS1606 shares 56.5% of its genome with Bacillus licheniformis, 18.7% with *Bacillus paralicheniformis,* and 17.6% with *Bacillus haynesii* (NCBI SRA Taxonomy Analysis Tool). A total of four genes of the auxin biosynthesis pathway and several protein–coding genes involved in the biocontrol process are present in the genome of this strain, thereby making it possible to use as BSt for enhancing agronomic applications. Similarly, in a recent study, the whole genome sequencing and root colonization analysis using a yellow fluorescence protein tag was conducted on Cucumber to understand the novel insights into the potential of *Bacillus subtilis* MBI 600 as biocontrol and growth promotion (Samaras et al., 2021). The above studies will assist in understanding the role and potential of such micro–organisms for use as BSts in agriculture.

Gene ontology analyses will help determine the genetic basis of plant adaptation and acclimation. Gene expression of key enzymes involved in synthesizing secondary metabolites or targeted physiological processes and transcriptome profiling to determine upregulated and downregulated genes will let us know about the expression of genes involved in stress response, adaptation, and important physiological processes related to the healthy growth of the plant. The expression can be studied along with epigenomics which deals with quantifying changes in epigenetic marks following environmental stress i.e., DNA methylation, post–translational histone modifications, and non–coding RNAs (siRNAs and miRNAs). Any change in epigenetic marks due to the effect of BSts and its correlation to the establishment of stable and transgenerational memory of stress response through plant BSts needs to be explored (Villagómez–Aranda et al., 2022). High–throughput omics tools, including phenomics, genomics, proteomics, metabolomics, and multi–omics approaches (Sahoo et al., 2022), can provide unique opportunities for precise decoding of the mode of action of BSts on the crop. For instance, Sudiro et al. (2022) performed phenomics investigations coupled with mass spectrometric untargeted metabolomics to unravel the mechanism behind drought tolerance through foliar applications of 4–Vita in tomatoes. A series of coordinated biochemical mechanisms could be detected in retort to the BSt treatment under drought conditions, including the modulation of thylakoid membrane lipids, the enhancement in the level of xanthins involved in ROS detoxification, and the chlorophyll synthesis supporting the high resilience of tomato to drought stress. Transcriptomics can be followed by genome editing techniques like CRISPR/Cas9 (Clustered Regulatory Interspaced Short Palindromic Repeats/CRISPR associated protein 9 system) to develop stress–resistant crops. Proteome analysis greatly helps understand the translated proteins involved in major physiological processes and enables the identification and characterization of novel proteins beneficial for plant growth and yield. Targeted metabolomics has dramatically enhanced our understanding of the effect of BSts on plant growth and stress tolerance by revealing the levels of various primary and secondary metabolites in treated plants. Using advanced computational and bioinformatics tools, we can get a clear picture of the metabolic pathways involved, which will help in developing/designing novel BSts. Recent studies have already proven that BSts can alter the expression of genes and metabolic pathways in alleviating various environmental stresses like heat and drought (**Supplementary Table 7**). Studying phenotypic changes involves a tremendous task, including measuring the various phenotypic characters at the cellular, organ, or whole plant level. The latest technologies available to measure large–scale phenotypic traits are advanced imaging systems, such as sensor–based systems, magnetic resonance imaging (MRI), and multispectral imaging systems (Rico–Chávez et al., 2022).

**Figure 2:**
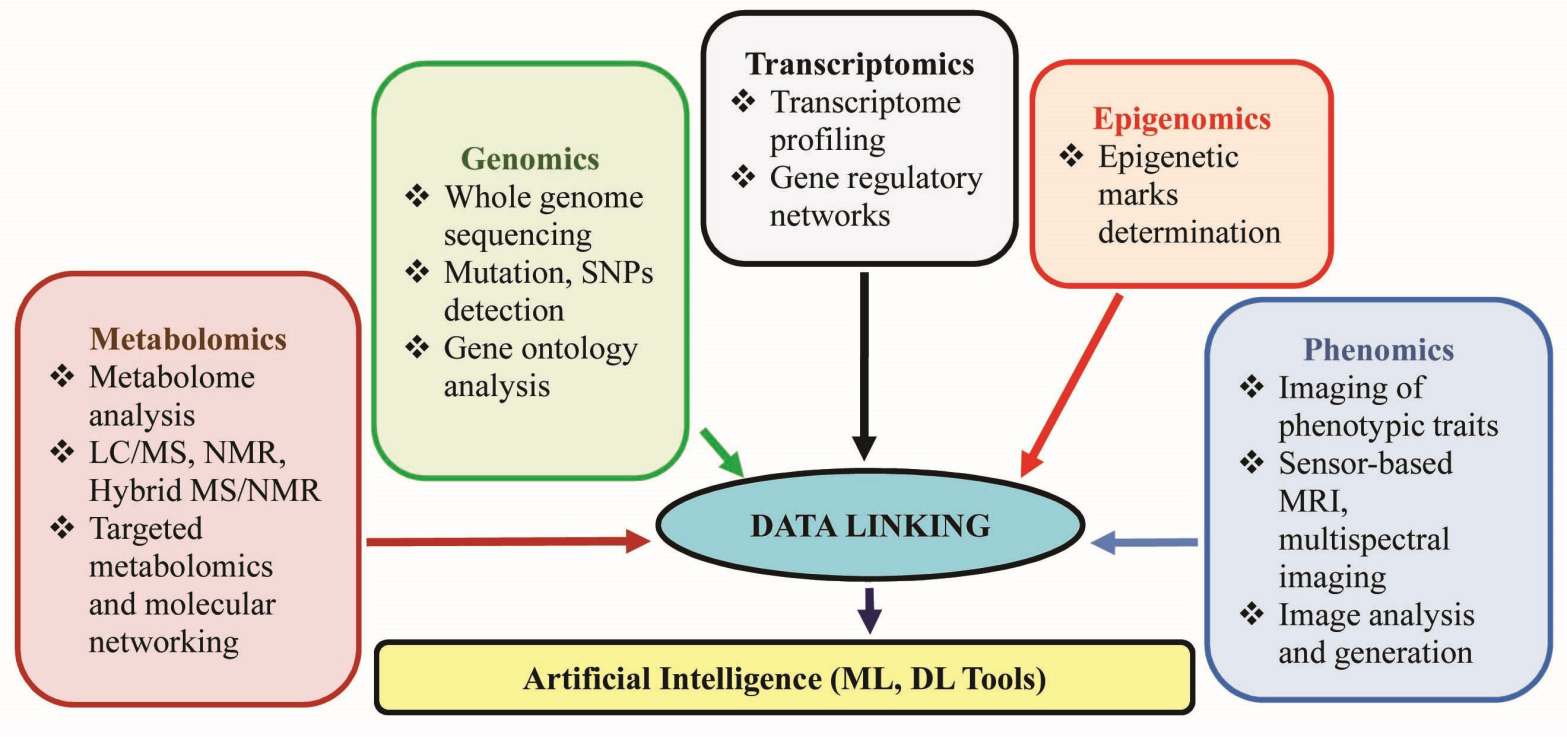
Multi–omics approach for understanding plant systems under stress and the role of biostimulants.

Although recent studies have demonstrated the significant contribution of the various “*omics*” approaches (especially transcriptomics, proteomics, and metabolomics) in providing insights into the mode of action of BSts, there are still a lot of limitations and challenges in each of the approaches. The gene expression level may not always correspond to the protein levels, and some proteins may not be functionally active. Therefore, transcriptomics and proteomics data singly cannot be referred to as the physiological status of a plant in response to BSts and abiotic stresses. The role of non–coding regions and regulatory elements cannot be ignored. These parts of the genome need to be explored in correlation with plant response to abiotic stresses and the effect of BSts to plant response. The metabolomics approach is yet another challenging field as it involves multiple steps, which are very crucial such as metabolite extraction, detection and data acquisition, data processing, and interpretation. To achieve fruitful research insights out of metabolomics study, a team with expertise in various fields like analytical chemistry, chemometrics, and data analytics is essential. A multi–*omics* approach can be followed to evaluate the role of BSts and develop strategies for a proper response along with the degree of environmental stress (**Figure 2**). With the advent of artificial neural networks technology, the large amount of data obtained using the various “*omics*” approaches can be processed by artificial intelligence (AI) techniques, mainly machine learning (ML) and deep learning (DL) tools, and use to identify, classify, or predict traits (Sahoo et al., 2022). We have discussed the significant influence of BSts accelerating rhizosphere microbiota, strengthening plant nutritional status, enhancing stress tolerance by producing secondary metabolites, and regulating gene and protein networks, which pave a future scope of microbiome engineering using advanced multi–*omics* tools.

## 6 Conclusion

Biostimulants have displayed a tremendous potential for mitigating the abiotic stressors that climate change has exacerbated without compromising crop production, productivity, and quality that will fulfil the food and nutritional security. These organic agro–inputs appear to be a promising alternative to synthetic protectants and provide a solution for creating highly sustainable and eco–friendly agricultural practices. However, it is imperative and necessary to understand and create awareness about the basic knowledge of these organic–based products amongst the small and marginal farmers. Moreover, additional strengthening of biostimulant products and specific crop–based research and development is urgent under the changing climate context. Assessing and validating biostimulants in certain aspects, like their efficacy and safety, is a present issue and concern because their performance varies in different crops under dissimilar environments. Modern approaches using high–throughput analyses, as in the various ‘*omics*’ approaches, could precisely validate the role of biostimulants and help in planning future strategies. Extensive investigations in this area could enable us to comprehend the underlying molecular mechanisms of biostimulants and how several reforms take place in metabolic pathways under harsh environmental conditions. A better understanding of the mode of action of biostimulation could further promote the valorization chain of biostimulants derived from waste products. Nowadays, there exist varying risk and efficacy assessment norms with unclear and inconsistent classifications around the globe. A harmonized international legislation, particularly regarding the registration and regulation of biostimulants, will further pave the way for easy access and use by the common masses. Overall, concerted efforts of government, non–government organizations, researchers, and other stakeholders will play a decisive role in eliminating all these constraints by working together from the grassroot level.

## Author contributions

IB, SKC, ELD, and RR: Conceptualization. SKC, ELD, RR, AKC, MDS, MRS, TLB, AST, and CB: Initial draft preparation. MRS, HMN, AK, MD, YPD, DS, SB, CPD, HRS, and CIK: Figures, tables, and editing. IB, SKC, ELD, and SHD: Critical review and finalization. All authors contributed to the article and approved the submitted version.

## Funding

The authors did not receive any sort of financial assistance from any funding agency or any organization. So you are requested to permit us the maximum APC if the Manuscript is accepted.

## Conflict of interest

The authors declare that the research was conducted in the absence of any commercial or financial relationships that could be construed as a potential conflict of interest.

## Publisher’s note

All claims expressed in this article are solely those of the authors and do not necessarily represent those of their affiliated organizations, or those of the publisher, the editors and the reviewers. Any product that may be evaluated in this article, or claim that may be made by its manufacturer, is not guaranteed or endorsed by the publisher.
