# Supplementary material for: Role of biostimulants in mitigating the effects of climate change on crop performance
Source: Front Plant Sci. 2022 Oct 21;13:967665. doi: 10.3389/fpls.2022.967665 (PMC9634556; doi:10.3389/fpls.2022.967665)
Supplement: Supplementary file 1 [file DataSheet_1.docx]

**Supplementary Table 1: Bacterial genera commonly used as biostimulants**

| **Bacterial genera** | **Gram Negative/Positive** | **Mode of Existence** |
| --- | --- | --- |
| *Rhizobium* | Gram-negative | Anaerobic, symbiotic |
| *Azospirillum* | Gram-negative | Anaerobic, free-living |
| *Pseudomonas* | Gram-negative | Aerobic but showed facultative anaerobic, free living |
| *Burkholderia* | Gram-negative | Aerobic, free-living |
| *Bacillus* | Gram-positive | Anaerobic or aerobic, free living |

**Supplementary Table 2: Various commonly used biostimulants and their role in nutrient absorption**

| **Biostimulants** | **Source** | **Crop** | **Role** | **References** |
| --- | --- | --- | --- | --- |
| Humic acid | Sludge of municipal waste | *In vitro* assay | - Inhibit urease enzyme activity in the soil | Liu et al. (2019) |
| Humic acid | Vermicompost | Mangosteen | - Modulate charge balance of cytosol - Increase membrane fluidity and permeability thereby increase the nutrient uptake - Increase in the activity of the pyrophosphatase enzyme (H+ -PPase) | Gomes et al. (2019) |
| Humic acid | Coal | Wheat | - Increase in micronutrient solubility by reducing pH of the soil | Khan et al. (2018) |
| Fulvic Acid | VitaLink Fulvic (Commercial formulation) | Legume | - Enhanced N uptake by increasing the number of root nodules in legumes | Capstaff et al. (2020) |
| Humic acid | Leonardite | Cucumber | - Increase activity of plasma membrane H+-ATPase, upregulation of CsFRO1 and CsIRT1 - Increase activity of Fe (III) chelate-reductase | Aguirre et al. (2009) |
| Sea weed extract | *Ascophyllum nodosum* | Lettuce | - Enhanced the relative growth rate of lettuce in the low-K treatment | Chrysargyris et al. (2018) |
| Seaweed sap | *Kappaphycus alvarezii* and *Gracilaria edulis* | Maize | - Increased the uptake of macro and minor nutrients | Basavaraja et al. (2018) |
| Seaweed sap | *Ascophyllum nodosum and Durvillea potatorum* | Tomato | - Mitigation of iron chlorosis | Carrasco-Gil et al. (2018) |
| Rosemary essential oil | Fresh rosemary foliage (steam-distillation) | Tomato | - Nutrient uptake and use efficiency (N, K, Mg, Fe and Zn) | Souri and Bakhtiarizade (2019) |
| Kelpak® and *Phomopsis columnaris* | *Ecklonia maxima* | *Noccaea goesingensis* | - Ni accumulation by 48% | Ważny et al. (2021) |

**Supplementary Table 3: Effect of salinity on plant growth and development**

| **Growth stage/ Plant Structure** | **Action/Consequences** | **References** |
| --- | --- | --- |
| Germination stage | Reduction in water imbibition by seed as a result of declined soil osmotic potential | Javaid et al. (2022); Khan and Weber (2008) |
|  | Change in the metabolism of protein | Rasheed (2009); Sarraf et al. (2022) |
|  | Significant decline in germination rate, plumule, root, and shoot length, and seed vigour | Khodarahmpour et al. (2012); Malik et al. (2022) |
| Plant physiology | Reduction in osmotic potential under high salinity | Gama et al. (2007); Javaid et al. (2022); Kaymakanova and Stoeva (2008); Rodriguez et al. (1997); |
|  | Decrease in plant water uptake, reduced turgidity in plant cells, declined cell division and regulation of stomata aperture, and consequently reduction in photosynthesis and ultimately death of plant tissues | Ali et al. (2021); Marschner (1995); Munns et al. (2002) |
|  | Altered membrane permeability, destabilized membrane proteins | Farouk et al. (2020); Grattan and Grieve (1992); Gupta et al. (2002) |
|  | Membrane interruption, nutrient imbalance, impaired ROS detoxification mechanisms, reduced photosynthesis, and reduced stomatal aperture | Rahnama et al. (2010); Shen et al. (2022) |
| Plant anatomy | Thicker leaves, epidermis, cell walls, and cuticles | Aslam et al. (2017) |
|  | Increased stomatal density on the lower side of leaves, amplified leaf thickness, palisade tissues, vascular bundle length, xylem rows, and reduced number of vessels | Hussein et al. (2012); Raafat et al. (1991) |
|  | Thickening of endo-as well as exodermis, lignification of intercellular spaces in the exodermis | Degenhardt and Gimmler (2000); Gomes et al. (2011); |
|  | Elevated suberin content in roots | Walker et al. (1985) |
| Plant morphology | Reduction in shoot growth, leaf development, and expansion, decreased internodal growth, and enhanced leaf abscission | Ali et al. (2021); Zekri (1991) |
|  | Dark green, thicker, and succulent leaves with declined leaf area and volume | Aslam et al. (2017); Bray and Reid (2002) |
|  | Diminished biomass and leaf area | Ashraf and Bhatti (2000); Challabathula et al. (2022) |

**Supplementary Table 4: Role of biostimulants in mitigating salt stress in crop plants**

| **Types of BS** | **Crop** | **Mechanisms** | **References** |
| --- | --- | --- | --- |
| Algal extract yield | Soybean (*Glycine max*) | Enhanced seed yield | Islam et al. (2021) |
| Humic acid extracts | Common bean (*Phaseolus vulgaris*), Rice (*Oryza sativa*) | Activation of anti-oxidative enzymatic function, enhanced ROS scavenging enzymes, detoxifying harmful free oxygen radicals synthesized, augmented endogenous proline levels and minimised membrane leakage in plants under drought and saline stress | Aydin et al. (2012); García et al. (2012) |
| Arbuscular mycorrhizal fungi (AMF) | Tomato (*Lycopersicon esculentum*) | Negating the salinity-induced oxidative stress | Abdel Latef and Chaoxing (2011) |
| Plant growth-promoting rhizobacteria | Wheat (*Triticum aestivum*) | Improved growth and yield | Upadhyay and Singh (2015) |
| Retrosal® (Valagro S.p.A) | Lettuce (*Lactuca sativa*) | Maintain the salinity level (NaCl) of water exposed, more accumulation of proline and less ABA content | Bulgari et al. (2019) |

**Supplementary Table 5: Physio-biochemical modulations of the plant by biostimulants**

| **Particular** | **Role/Action** | **References** |
| --- | --- | --- |
| **Ionic compartmentalization and homeostasis** | | |
| Proline | Higher accumulation of proline in antioxidant defense mechanisms improves salt tolerance | Hoque et al. (2007); Meena et al., (2019) |
| Na+/Cl- ratio | A high concentration of Na^+^ is toxic for the plant cell causing a low metabolic rate for the plant, destabilization of the membrane, and cell division becoming slow finally changing the homeostasis of the mineral nutrients | Munns and Tester, (2008) |
|  | Regular control of Na^+^ entry in the cytoplasm of the cell through Na^+^/H^+^ antiporter *via* vacuolar-type H^+^-ATPase (V-ATPase) and the vacuolar pyrophosphatase (V-PPase) and its compartmentalization into the vacuole maintain the ionic homeostasis in plants | Mansour (2022) |
|  | Na^+^ and Cl^-^ ion concentration within the cell, maintains the growth of the plant | Assaha et al. (2017); Hasegawa et al., (2000) |
| **Osmotic adjustment** | | |
| Compatible solute | More production of polyols, glycine betaine, and soluble sugar maintain the required osmotic potential inside the cell | Kerepesi and Galiba (2000) |
|  | Compatible solute maintains the cell's pH, detoxified harmful chemicals, and free radicles produced during the oxidation process are clean and | Mansour et al. (2000); Slama et al. (2015) |
|  | Reduction of osmotic potential due to excess accumulation of compatible salt | Hasegawa et al. (2000) |
| Proline | During the stress condition proline serves as a reserve organic nitrogen to recover the stress,  pyrroline carboxylic acid synthetase and pyrroline carboxylic acid reductase play a vital role to produce proline from the primary precursor glutamate in the biosynthetic pathway | Qamar et al. (2015) |
|  | Increasing the enzyme activity responsible for the production of proline in antioxidant defense mechanisms improves salt tolerance | Hoque et al. (2007);  Meena et al. (2019) |
| **Antioxidants** | | |
| Glutathione | ROS scavenging, stress-responsive gene expression, detoxification of xenobiotics | Massange-Sánchez et al. (2021); Mullineaux and Rausch (2005); Noctor et al. (2012) |
| Tocopherol | Reduced lipid peroxidation, cell signaling, regulation of growth regulation phytohormones | Krieger-Liszkay and Trebst (2006); Maeda et al. (2006); Munné-Bosch and Alegre (2002) |
| Carotenoids | Heat dissipation, protection of photosynthetic apparatus, regulation of hormonal biosynthesis | Jin et al. (2015); Ruiz-Sola and Rodríguez-Concepción (2012) |
| Flavonoids | Inhibition of ROS-producing enzymes; antioxidant barriers | Agati et al., (2012); Mierziak (2014) |
| Ascorbic acid | ROS scavenging, Heat dissipation, synthesis of growth regulating phytohormones, induction of cell signaling, and cell division | Gallie (2013); Massange-Sánchez et al. (2021); Smirnoff (2000) |
| **Other compounds** | | |
| Silicon | Si improves plant growth either directly by blocking the transport of Na^+^ ions into the plant, or indirectly by activating different physiological processes to ameliorate the effect of salinity stress | Dhiman et al. (2021) |
| Helicase proteins (DESD-box helicase and OsSUV3 dual helicase) | Maintains or improves photosynthesis and antioxidant enzyme machinery | Tuteja et al. (2013) |

**Supplementary Table 6: Different biostimulants used in mitigating temperature stress**

| **Biostimulants** | **Crop** | **Mode of application** | **Role** | **References** |
| --- | --- | --- | --- | --- |
| **High temperature stress** | | | | |
| CycoFlow (sugar cane molasses with yeast extract) | Tomato | Fertigation | Increased ascorbic acid, pollen viability, and antioxidant activity | Francesca et al. (2020) |
| Boosten and Megafol derived from seaweed,  Sabion derived from animal collagen | Tomato | Foliar application | CO_2_ assimilation by stabilizing chlorophyll pigments Improved root growth aid in nutrient uptake | Niu et al. (2022) |
| Seaweed extract | Wheat | Seed pre-treatment and fertigation | Increased antioxidant capacity and chlorophyll stability | Jócsák et al. (2022) |
| KIEM^®^ (2% organic nitrogen, 2% molybdenum, and 21% organic carbon) | Soybean | Seed priming | Increased germination percentage, upregulation genes involved in DNA repair, protection from enzymatic degradation and methyl transferases.  Reduced oxidative stress | Campobenedetto et al. (2020) |
| Salicylic acid (SA)  Jasmonic acid (JA)  Nitric oxide (NO) as sodium nitroprusside (SNP) | Kimchi cabbage (*Brassica rapa* L. *ssp. pekinensis*) | Foliar application | Reduced the transpiration up to 50% compared to only heat-treated plants, biostumilant able to maintain higher rate of photosynthesis and yield under heat stress | Lee et al. (2019) |
| Protein hydrolysate (CycoFlow) | Tomato | Soil application | Reduced H_2_O_2_ accumulation  and MDA content and increased antioxidant content | Francesca et al. (2022) |
| Humic acid along with *Bacillus cereus* | Tomato | Soil inoculation | Reduced ABA and increased SA on contrary to non-stressed plants, Enhanced the stability of PSII under heat stress | Khan et al. (2020a) |
| **Low temperature Stress** | | | | |
| Smoke-water and KAR_1_ | *Ceratotheca triloba* | Seed priming | Improved germination by mitigating detrimental effects of low temperature | Masondo et al., (2018) |
| Asahi SL (synthetic biostimulant)  Goëmar Goteo (extract of *Ascophyllum nodosum*) | *Coriandrum sativum* | Foliar application | Increased rate of transpiration and stomatal conductance maximum, quantum yield of PSI | Pokluda et al., (2016) |
| Ruter AA, Terra Sorb and Razormin | Rapeseed and Wheat | Foliar spray | More accumulation of free amino acids, (most probably proline and glutamine) | Gaveliene et al. (2018) |

**Table 7: Study cases using modern methods of analysis of biostimulants on different plants under stress**

| **S. No.** | **Study** | **Methods used** | **Stress condition** | **Inference** | **Reference** |
| --- | --- | --- | --- | --- | --- |
| 1 | EnNuVi® ALPAN® on tomato; foliar application | Transcriptome studies (RNA-seq and qPCR); qualitative analyses | Drought | Upregulation of drought-responsive genes; stabilization of photosynthetic pigment levels | Hamedeh et al. (2022) |
| 2 | Seaweeds extract on maize; foliar application | Metabolomics and molecular networking; LC-MS and computational tools; Physiological studies | Drought | Alteration in primary and secondary metabolic pathways | Tinte et al., (2022) |
| 3 | Microbial consortia on maize; soil application | Metabolomics and molecular networking; LC-MS and bioinformatics tools; Biochemical analysis of metabolites | Drought | Alteration in amino acids profile, TCA intermediates, phenolics, and hormones involved in various pathways | Othibeng et al. (2022) |
| 4 | Phylgreen (*Ascophyllum nodosum* extracts) and Delfan Plus (animal L-α amino acids) on *Arabidopsis thaliana* plants; foliar application | Transcriptome studies, physiological, and biochemical analyses |  | Upregulation of heat stress response genes and antioxidant systems, reduction of oxidative damage in leaf morphology | Cocetta et al. (2022) |
| 5 | Calcium based biostimulant on tomato; foliar application | Transcriptomics and physiological profiling, RT-qPCR (real-time quantitative reverse transcription PCR) | Water-deficient | Expression of 9 genes involved in nutrient metabolism and osmotic stress were validated; increased photosynthetic rate and chlorophyll content | Rico-Chávez et al. (2022) |
| 6 | Protein hydrolysates on *Arabidopsis thaliana*; seed priming | High throughput screening and untargeted metabolomics; physiological analyses | High salt | Reduced content of stress-related molecules, Increased photosynthetic rates | Sorrentino et al. (2021) |
| 7 | *Ascophyllum nodosum* extracts | Transcriptomics, physical, biochemical analyses | Heat | Expression of heat shock proteins, increased flower development, fruit production, soluble sugars accumulation | Carmody et al. (2020) |

**References (excluding those in the manuscript)**

Abdel Latef, A. A. H., and Chaoxing H. (2011). Arbuscular mycorrhizal influence on growth, photosynthetic pigments, osmotic adjustment and oxidative stress in tomato plants subjected to low temperature stress. *Acta Physiol. Plant*., 33, 1217-1225.

Agati, G., Azzarello, E., Pollastri, S., and Tattini, M. (2012). Flavonoids as antioxidants in plants: Location and functional significance. *Plant Sci.* 196, 67-76

Aguirre, E., Leménager, D., Bacaicoa, E., Fuentes, M., Baigorri, R., Zamarreño, A. M., et al. (2009). The root application of a purified leonardite humic acid modifies the transcriptional regulation of the main physiological root responses to Fe deficiency in Fe-sufficient cucumber plants. *Plant Physiol. Biochem.* *47*(3), 215–223. <https://doi.org/10.1016/j.plaphy.2008.11.013>

Ashraf, M. Y., and Bhatti, A. S. (2000). Effect of salinity on growth and chlorophyll content in rice. *Pak. J. Sci. Ind. Res*. 43, 130-131.

Aslam M., Ahmad K., Akhtar M. A., and Maqbool M. A. (2017). Salinity stress in crop plants: Effects of stress, tolerance mechanisms and breeding strategies for improvement. *J. Agric. Biol. Sci.* 2(1), 70-85.

Assaha, D. V. M., Ueda, A., Saneoka, H., Al-Yahyai, R., and Yaish, M. W. (2017). The role of Na+ and K+ transporters in salt stress adaptation in glycophytes. *Front Physiol*. 18(8), 509. doi: 10.3389/fphys.2017.00509.

Aydin, A., Kant, C., and Turan, M. (2012).  Humic acid application alleviates salinity stress of bean (*Phaseolus vulgaris* L.) plants decreasing membrane leakage. *Afric. J. of Agri. Res*. 7, 1073-1086. doi: 10.5897/ ajar10.274

Basavaraja, P. K., Yogendra, N. D., Zodape, S. T., Prakash, R., and Ghosh, A. (2018). Effect of seaweed sap as foliar spray on growth and yield of hybrid maize.  
*J. Plant Nutr.* 41(14), 1851-1861.

Bray, S., and Reid, D. M. (2002). The effect of salinity and CO_2_ enrichment on the growth and anatomy of the second trifoliate leaf of *Phaseolus vulgaris*. *Can. J. Bot*. 80, 349-359.

Bulgari, R., Trivellini, A., and Ferrante, A. (2019). Effects of two doses of organic extract-based biostimulant on greenhouse lettuce grown under increasing NaCl concentrations. *Front.Plant Sci.*, 9, 1–14. <https://doi.org/10.3389/fpls.2018.01870>

Carmody, N., Goñi, O., Łangowski, Ł., and O’Connell, S. (2020). Ascophyllum nodosum extract biostimulant processing and its impact on enhancing heat stress tolerance during tomato fruit set. *Front.Plant Sci.*, 11, 807.

Carrasco-Gil, S., Hernandez-Apaolaza, L., and Lucena, J. J. (2018). Effect of several commercial seaweed extracts in the mitigation of iron chlorosis of tomato plants (*Solanum lycopersicum L*.). *Plant growth regul.* 86(3), 401-411.

Challabathula, D., Analin, B., Mohanan, A., and Bakka, K. (2022). Differential modulation of photosynthesis, ROS and antioxidant enzyme activities in stress-sensitive and -tolerant rice cultivars during salinity and drought upon restriction of COX and AOX pathways of mitochondrial oxidative electron transport. *J. Plant Physiol*. 268. [https://doi.org/10.1016/j.jplph.2021.153583](about:blank)

Chrysargyris, A., Xylia, P., Anastasiou, M., Pantelides, I., and Tzortzakis, N. (2018). Effects of *Ascophyllum nodosum* seaweed extract on lettuce growth, physiology and fresh-cut salad storage under potassium deficiency. *J. Sci. Food Agric.* 98(15), 5861–5872. <https://doi.org/10.1002/jsfa.9139>

Degenhardt, B., and Gimmler, H. (2000). Cell wall adaptations to multiple environments stresses in maize root. *J. Exp. Bot*. 51, 595-603.

Dhiman, P., Rajora, N., Bhardwaj, S., Sudhakaran, S. S., Kumar, A., Raturi, G., et al. (2021). Fascinating role of silicon to combat salinity stress in plants: An updated overview. *Plant Physiol. Biochem*. 162, 110-123. https://doi.org/10.1016/j.plaphy.2021.02.023.

Farouk, S. S., Elhindi, K. M. and Alotaibi, M. A. (2020). Silicon supplementation mitigates salinity stress on *Ocimum basilicum* L. via improving water balance, ion homeostasis, and antioxidant defense system. *Ecotoxicol. Environ. Saf*. 206, 11.

Francesca, S., Arena, C., Hay Mele, B., Schettini, C., Ambrosino, P., Barone, A., et al. (2020). The use of a plant-based biostimulant improves plant performance and fruit quality in tomato plants grown at elevated temperatures. *Agronomy* 10(3), 363.

Francesca, S., Najai, S., Zhou, R., Decros, G., Cassan, C., Delmas, F., et al. (2022). Phenotyping to dissect the biostimulant action of a protein hydrolysate in tomato plants under combined abiotic stress. *Plant Physiol. Biochem.*, 179, 32-43.

Gallie, D. R. (2013). L-Ascorbic acid: A multifunctional molecule supporting plant growth and development. *Scientifica*. Article ID 795964 | https://doi.org/10.1155/2013/795964

Gama, P. B. S., Inanaga, S., Tanaka, K., and Nakazawa, R. (2007). Physiological response of common bean (*Phaseolus vulgaris* L.) seedlings to salinity stress. *Afr. J. Biotechnol*. 6,79-88.

García, A. C., Santos, L. A., Izquierdo, F. G., Sperandio, M. V. L., Castro, R. N., and Berbara R. L. L. (2012).  Vermicompost humic acids as an ecological pathway to protect rice plant against oxidative stress. *Eco. Engineering*. 47, 203-208. doi: 10.1016/j. ecoleng.2012.06.011

Gaveliene, V., L. Pakalniskyte, L. Novickiene, and L. Balciauskas. (2018). Effect of Biostimulants on Cold Resistance and Productivity Formation in Winter Rapeseed and Winter Wheat. *Irish J. Agric. Food Res.* 57, 71–83. <https://www.jstor.org/stable/26555013>.

Gomes, G. A., Pereira, R. A., Sodré, G. A., and Gross, E. (2019). Humic acids from vermicompost positively influence the nutrient uptake in mangosteen seedlings. *Pesqui. Agropecu. Trop.* 49. https://doi.org/10.1590/1983-0632019v4955529

Gomes, M. P., de SA e Melo Marques, T.C.L.L., de Oliveira Goncalves Nogueira, M., de Castro, E. M., and Soares, A. M. (2011). Ecophysiological and anatomical changes due to uptake and accumulation of heavy metal in *Brachiaria decumbens*. *Sci. Agric.* 68(5), 566-573. https://doi.org/10.1590/S0103-90162011000500009

Grattan, S. R., and Grieve, C. M. (1992). Mineral element acquisition and growth response of plants grown in saline environment. *Agric. Ecosyst. Environ.* 38, 275-300.

Gupta, I. K., Meena, S. K., Gupta, S., and Khandelwal, S. K. (2002). Gas exchange, membrane permeability, and ion uptake in two species of Indian *jajuba* differing in salt tolerance. *Photosynthetica* 40, 535-53.

Hasegawa, P. M., Bressan, R. A., Zhu, J. K. and Bohnert, H. J. (2000). Plant cellular and molecular responses to high salt. *Annu. rev. plant physiol. plant mol. biol.* 51, 463– 499.

Hussein, M.M., Abo-Leila, B.H., Metwally, S. A., and Leithy, S. Z. (2012). Anatomical structure of *jatropha* leaves affected by proline and salinity conditions. *J. Appl. Sci. Res.* 8, 491-496.

Islam, M.T., Ckurshumova, W., Fefer, L. M., Uddin, J., and Rosa, C. (2021). A plant based modifified biostimulant (*Copper Chlorophyllin*), mediates defence response in *Arabidopsis thaliana* under salinity stress. *Plants* 10, 625.

Javaid, M. M., Mahmood, A., Alshaya, D. S.,  AlKahtani, M. D. F., Waheed, H., Wasaya, A., et al. (2022). Influence of environmental factors on seed germination and seedling characteristics of perennial ryegrass (*Lolium perenne* L.). *Sci. Rep.* 12, 9522. [https://doi.org/10.1038/s41598-022-13416-6](about:blank)

Jin, C., Ji, J., Zhao, Q., Ma, R., Guan, C., and Wang, G. (2015). Characterization of lycopene β-cyclase gene from *Lycium chinense* conferring salt tolerance by increasing carotenoids synthesis and oxidative stress resistance in tobacco. *Mol Breed*. 35(12), 228.

Jócsák, I., Gyalog, H., Hoffmann, R., and Somfalvi-Tóth, K. (2022). *In-Vivo* Biophoton emission, physiological and oxidative responses of biostimulant-treated winter wheat (*Triticum eastivum* L.) as seed priming possibility, for heat stress alleviation. *Plants*, 11(5), 640.

Kaymakanova, M., and Stoeva, N. (2008). Physiological reaction of bean plants (*Phaseolus vulgares* L.) to salinity stress. *Gen. Appl. Plant Physiol.* 34, 177-188.

Kerepesi, I., and Galiba, G. (2000). Osmotic and salt stress-induced alteration in soluble carbohydrate content in wheat seedlings. *Crop Sci.* 40(2). DOI: 10.2135/cropsci2000.402482x

Khan, M. A., and Weber, D. J. (2008). Ecophysiology of high salinity tolerant plants. (Tasks for Vegetation Science). *Series*. Springer, Amsterdam

Khodarahmpour, Z., Ifar. M., and Motamedi, M. (2012). Effects of NaCI salinity on maize (*Zea mays* L.) at germination and early seedling stage. *Afr. J. Biotechnol*. 11,298-304.

Krieger-Liszkay, A., and Trebst, A. (2006). Tocopherol is the scavenger of singlet oxygen produced by the triplet states of chlorophyll in the PSII reaction centre. *J. Exp. Bot.* 57(8), 1677-84. DOI:0.1093/jxb/erl002

Lee, H. J., Lee, J. H., Lee, S. G., An, S., Lee, H. S., Choi, C. K., et al. (2019). Foliar application of biostimulants affects physiological responses and improves heat stress tolerance in Kimchi cabbage. *Hortic. Environ. Biotechnol*. 60(6), 841-851.

Liu, H., Yin, H., Tang, S., Wei, K., Peng, H., Lu, G., et al. (2019).Effects of benzo [a] pyrene (BaP) on the composting and microbial community of sewage sludge. *Chemosphere*, 222, 517-526. 10.1016/j.chemosphere.2019.01.180

Maeda, H., Song, W., Sage, T. L., and DellaPenna, D. (2006). Tocopherols play a crucial role in low-temperature adaptation and phloem loading in *Arabidopsis*. *Plant Cell.* 18(10), 2710-2732.

Malik, J. A., AlQarawi, A. A., AlZain, M. N., Dar, B. A., Habib, M. M., and Ibrahim, S.N.S. (2022). Effect of salinity and temperature on the seed germination and seedling growth of desert forage grass *Lasiurus scindicus* Henr. *Sustainability* 14, 8387.

Mansour, M. M. F. (2000). Nitrogen containing compounds and adaptation of plants to salinity stress. *Biol. Plant.* 43, 491–500.

Mansour, M. M. F. (2022). Role of vacuolar membrane transport systems in plant salinity tolerance. *J. Plant Growth Regul*. https://doi.org/10.1007/s00344-022-10655-9

Marschner, H. (1995). Part I. Nutritional physiology. In: Marschner, H. (Eds.) Mineral nutrition of higher plants. Academic Press, London. Second ed.18-30, 313-363.

Masondo, N. A., Kulkarni, M. G., Finnie, J. F., and Van Staden, J. (2018). Influence of biostimulants-seed-priming on Ceratotheca triloba germination and seedling growth under low temperatures, low osmotic potential and salinity stress. *Ecotoxicol. Environ. Saf.*, 147, 43-48.

Massange-Sánchez, J. A., Sánchez-Hernández, C. V., Hernández-Herrera, R. M., and Palmeros-Suárez, P. A. (2021). The biochemical mechanisms of salt tolerance in plants. In Hasanuzzaman, M. and Nahar, K. (Eds.), Plant Stress Physiology - Perspectives in Agriculture. IntechOpen. https://doi.org/10.5772/intechopen.101048

Meena, M., Divyanshu, K., Kumar, S., Swapnil, P., Zehra, A., Shukla. V., et al. (2019). Regulation of L-proline biosynthesis, signal transduction, transport, accumulation and its vital role in plants during variable environmental conditions. *Heliyon* 5(12), e02952. https://doi.org/10.1016/j.heliyon.2019.e02952.

Mierziak, J., Kostyn, K., and Kulma, A. (2014). Flavonoids as important molecules of plant interactions with the environment. *Molecules* 19(10),16240-16265.

Mullineaux, P. M., and Rausch, T. (2005). Glutathione, photosynthesis and the redox regulation of stress-responsive gene expression. *Photosynth Res*. 86(3), 459-474. DOI:10.1007/s11120-005-8811-8

Munné-Bosch, S., and Alegre, L. (2002). The function of tocopherols and tocotrienols in plants. *CRC Crit. Rev. Plant Sci.* 21(1), 31-57. DOI:10.1080/0735-260291044179

Munns, R., and Tester, M. (2008). Mechanisms of salinity tolerance. *Annu. Rev. Plant Biol.* 59, 651– 681.

Niu, C., Wang, G., Sui, J., Liu, G., Ma, F., and Bao, Z. (2022). Biostimulants alleviate temperature stress in tomato seedlings. *Sci. Hortic*. 293, 110712.

Noctor, G., Mhamdi, A., Chaouch, S., Han, Y. I., Neukermans, J., Márquez-García, B., et al. (2012). Glutathione in plants: an integrated overview. *Plant Cell Environ*. 35(2), 454-484. DOI:10.1111/j.1365-3040.2011.02400.x

Othibeng, K., Nephali, L., Myoli, A., Buthelezi, N., Jonker, W., Huyser, J., and Tugizimana, F. (2022). Metabolic circuits in sap extracts reflect the effects of a microbial biostimulant on maize metabolism under drought conditions. *Plants (Basel)*. 11(4):510. doi: 10.3390/plants11040510

Pokluda, R., Sękara, A., Jezdinský, A., Kalisz, A., Neugebauerová, J., and Grabowska, A. (2016). The physiological status and stress biomarker concentration of *Coriandrum sativum* L. plants subjected to chilling are modified by biostimulant application. *Biol. Agric. Hortic.*, 32(4), 258-268.

Qamar, A., Mysore, K. S. and Senthil-Kumar, M. (2015). Role of proline and pyrroline-5-carboxylate metabolism in plant defense against invading pathogens. *Front. Plant Sci*. <https://doi.org/10.3389/fpls.2015.00503>

Raafat, A., Habib, S. A., El-Shami, I. Z., and El-Antably, H. M. (1991). The effect of salinity on the anatomical features of tomato plants. *Ann. Agric. Sci*. 36, 307-321.

Rahnama, A., James, R. A., Poustini, K., and Munns, R. (2010). Stomatal conductance as a screen for osmotic stress tolerance in durum wheat growing in saline soil. *Funct. Plant Biol*. 37(3), 255–263.

Rasheed, R. (2009). Salinity and extreme temperature effects on sprouting buds of sugarcane (*Saccharum officinarum* L.). Some histological and biochemical studies. Ph.D. thesis Department of Botany, University of Agriculture, Faisalabad, Pakistan.

Rico-Chávez, A. K., Franco, J. A., Fernandez-Jaramillo, A. A., Contreras-Medina, L. M., Guevara-González, R. G., and Hernandez-Escobedo, Q. (2022). Machine learning for plant stress modeling: A perspective towards hormesis management. *Plants (Basel)*. 11(7):970. doi: 10.3390/plants11070970.

Rodriguez, P., Amico, J. D., Morales, D., Blanco, M. J. S., and Alarcon, J. J. (1997). Effects of salinity on growth, shoot water relations and root hydraulic conductivity in tomato plants*. J. Agric. Sci*. 128, 439-444.

Ruiz-Sola, M. Á., and Rodríguez-Concepción M. (2012). Carotenoid biosynthesis in Arabidopsis: a colorful pathway. *Arab B*. 10, e0158–e0158.

Sarraf, M., Vishwakarma, K., Kumar, V., Arif, N., Das, S., Johnson, R., et al. (2022). Metal/Metalloid-based nanomaterials for plant abiotic stress tolerance: An overview of the mechanisms. *Plants* 11, 316. [https://doi.org/10.3390/plants11030316](about:blank)

Shen, Z., Cheng, X., Li, X., Deng, X., Dong, X., Wang, S., et al. (2022). Effects of silicon application on leaf structure and physiological characteristics of *Glycyrrhiza uralensis* Fisch. and *Glycyrrhiza inflata* Bat. under salt treatment. *BMC Plant Biol.* 22, 390. [https://doi.org/10.1186/s12870-022-03783-7](about:blank)

Slama, I., Abdelly, C., Bouchereau, A., Flowers, T., and Savouré, A. (2015) Diversity, distribution and roles of osmoprotective compounds accumulated in halophytes under abiotic stress. *Ann. Bot*. 115(3), 433-447. doi: 10.1093/aob/mcu239.

Smirnoff, N. (2000). Ascorbate biosynthesis and function in photoprotection. *Phil. Trans. R. Soc. Lond B.* 355(1402), 1455-1464.

Sorrentino, M., De Diego, N., Ugena, L., Spíchal, L., Lucini, L., Miras-Moreno, B., et al. (2021). Seed priming with protein hydrolysates improves Arabidopsis growth and stress tolerance to abiotic stresses. *Front Plant Sci*. 12:626301. doi: 10.3389/fpls.2021.626301

Souri, M. K., and Bakhtiarizade, M. (2019). Biostimulation effects of rosemary essential oil on growth and nutrient uptake of tomato seedlings. *Sci. Hortic*. 243, 472-476.

Tinte, M. M., Masike, K., Steenkamp, P.A., Huyser, J., van der Hooft, J. J. J., and Tugizimana, F. (2022). Computational metabolomics tools reveal metabolic reconfigurations underlying the effects of biostimulant seaweed extracts on maize plants under drought stress conditions. *Metabolites* 12(6),487. doi: 10.3390/metabo12060487

Tuteja, N., Sahoo, R. K., Garg, B., and Tuteja, R. (2013). OsSUV3 dual helicase functions in salinity stress tolerance by maintaining photosynthesis and antioxidant machinery in rice (*Oryza sativa* L. cv. IR64). *Plant J*. 76(1), 115-127. doi: 10.1111/tpj.12277.

Upadhyay, S. K., and Singh, D. P. (2015). Effect of salt-tolerant plant growth-promoting rhizobacteria on wheat plants and soil health in a saline environment. *Plant Biol*. 17(1), 288-293. doi: 10.1111/plb.12173.

Walker, C. D., Graham, R. D., Madison, J. T., Cary, E. E., and Welch, R. M. (1985). Effects of Ni deficiency on some nitrogen metabolites in cowpea (*Vigna unguiculata* L. Walp). *Plant Physiol*. 79,474-479.

Ważny, R., Rozpądek, P., Jędrzejczyk, R. J., Domka, A., Nosek, M., Kidd, P., et al. (2021). Phytohormone based biostimulant combined with plant growth promoting endophytic fungus enhances Ni phytoextraction of Noccaea goesingensis. *Sci. Total Environ.* 789 147950.

Zekri, M. (1991). Effects of NaCl on growth and physiology of sour orange and *Cleopatra mandarin* seedlings. *Sci. Hortic*. 47, 305-315.
